# Lectures on Tumors from a Clinical Standpoint

**Published:** 1891-11

**Authors:** 


Lectures on Tumors, from a Clinical Standpoint. By John B. Hamilton, M. D., LL. D., Professor of Principles of Surgery and Clinical Surgery, Rush Medical College, Chicago, and in the Chicago Polyclinic ; formerly Supervising Surgeon-General U. S. Marine Hospital Service, etc., etc. For the use of students. *Physicians' Leisure Library*. Detroit : George S. Davis. 1891. Price, cloth, 50 cents ; paper, 25 cents.

The author of this little work is an experienced teacher and a thorough student. He is also one of the foremost surgeons of the land, and whatever he says will command attention because of this fact. It is important for a practitioner in the study of tumors, to have a guide that will enable him to follow out intelligently their clinical history. Very few are able to classify them properly in their own mind, hence find difficulty in interpreting their clinical manifestations correctly. Dr. Hamilton is not satisfied with the nomenclature and classification which he is obliged to follow, but still claims that is better than none, and must be adhered to until the next revision by the constituted authorities. The present one was adopted by the Royal College of Surgeons of London, and the American Medical Association, jointly, in 1880. Another revision will soon be ready for utilization, and we presume future editions of this book will conform to the modifications proposed. There are some errors in typography, which also then may be corrected, but taken altogether it is one of the clearest expositions on this difficult subject that has yet appeared, and its price is such as to bring it within the means of every physician. There need be no excuse hereafter for want of knowledge on this subject.

